# Dose accumulation for MR-guided adaptive radiotherapy: From practical considerations to state-of-the-art clinical implementation

**DOI:** 10.3389/fonc.2022.1086258

**Published:** 2023-01-26

**Authors:** Brigid A. McDonald, Cornel Zachiu, John Christodouleas, Mohamed A. Naser, Mark Ruschin, Jan-Jakob Sonke, Daniela Thorwarth, Daniel Létourneau, Neelam Tyagi, Tony Tadic, Jinzhong Yang, X. Allen Li, Uffe Bernchou, Daniel E. Hyer, Jeffrey E. Snyder, Edyta Bubula-Rehm, Clifton D. Fuller, Kristy K. Brock

**Affiliations:** ^1^ Department of Radiation Oncology, The University of Texas MD Anderson Cancer Center, Houston, TX, United States; ^2^ Department of Radiotherapy, University Medical Center Utrecht, Utrecht, Netherlands; ^3^ Elekta AB, Stockholm, Sweden; ^4^ Department of Radiation Oncology, University of Toronto, Sunnybrook Health Sciences Centre, Toronto, ON, Canada; ^5^ Department of Radiation Oncology, The Netherlands Cancer Institute, Amsterdam, Netherlands; ^6^ Section for Biomedical Physics, Department of Radiation Oncology, University of Tuebingen, Tuebingen, Germany; ^7^ Radiation Medicine Program, Princess Margaret Cancer Centre, Toronto, ON, Canada; ^8^ Department of Radiation Oncology, University of Toronto, Toronto, ON, Canada; ^9^ Department of Medical Physics, Memorial Sloan-Kettering Cancer Center, New York, NY, United States; ^10^ Department of Radiation Physics, The University of Texas MD Anderson Cancer Center, Houston, TX, United States; ^11^ Department of Radiation Oncology, Medical College of Wisconsin, Milwaukee, WI, United States; ^12^ Laboratory of Radiation Physics, Department of Oncology, Odense University Hospital, Odense, Denmark; ^13^ Department of Clinical Research, University of Southern Denmark, Odense, Denmark; ^14^ Department of Radiation Oncology, University of Iowa Hospitals and Clinics, Iowa City, IA, United States; ^15^ Department of Imaging Physics, The University of Texas MD Anderson Cancer Center, Houston, TX, United States

**Keywords:** dose accumulation, MR-guided radiation therapy, adaptive radiation therapy, deformable image registration, MR-linac

## Abstract

MRI-linear accelerator (MR-linac) devices have been introduced into clinical practice in recent years and have enabled MR-guided adaptive radiation therapy (MRgART). However, by accounting for anatomical changes throughout radiation therapy (RT) and delivering different treatment plans at each fraction, adaptive radiation therapy (ART) highlights several challenges in terms of calculating the total delivered dose. Dose accumulation strategies—which typically involve deformable image registration between planning images, deformable dose mapping, and voxel-wise dose summation—can be employed for ART to estimate the delivered dose. In MRgART, plan adaptation on MRI instead of CT necessitates additional considerations in the dose accumulation process because MRI pixel values do not contain the quantitative information used for dose calculation. In this review, we discuss considerations for dose accumulation specific to MRgART and in relation to current MR-linac clinical workflows. We present a general dose accumulation framework for MRgART and discuss relevant quality assurance criteria. Finally, we highlight the clinical importance of dose accumulation in the ART era as well as the possible ways in which dose accumulation can transform clinical practice and improve our ability to deliver personalized RT.

## 1 Introduction

In the current era of image-guided radiation therapy (RT), many technological advances in on-board imaging systems and treatment delivery techniques have enabled the delivery of highly conformal RT (1, 2). One major development in recent years has been the integration of magnetic resonance imaging (MRI) with linear accelerators (linacs) to form hybrid systems known as MR-linacs (3–5). MRI offers enhanced visualization of both tumor and normal tissue structures compared to other on-board imaging systems such as kV or MV planar x-ray, computed tomography (CT), and cone beam CT (6). The ability to clearly visualize the anatomy during patient setup has accelerated the development of on-line adaptive RT (ART), in which a new treatment plan is created each day based on the patient’s daily setup image while the patient is on the treatment table (7–9).

Daily MR-guided ART (MRgART) offers many dosimetric advantages over the traditional single-plan RT workflow, including the ability to conform the high-dose region to the tumor as the anatomy changes throughout the course of RT (9, 10). However, MRgART brings to the forefront a number of challenges in terms of calculating and interpreting the delivered dose that have largely been ignored in the past. In contrast to conventional RT, where dose estimates are calculated on a single pre-treatment simulation image, ART uses multiple plans created on longitudinal images reflecting anatomical variations throughout the treatment course. Currently, most clinical implementations of ART simply create new plans meeting the original treatment constraints and do not use advanced dose accumulation strategies to sum the doses from individual plans and account for these anatomical changes. Without using deformable image registration to truly sum the dose, the contributions of individual plans cannot be interpreted in the context of the total delivered dose and statements regarding over- or under-dosage of tissues cannot be accurately made. The ability to accurately quantify delivered dose allows clinicians to evaluate whether dosimetric criteria are being met in aggregate over multiple fractions and enables adaptation throughout RT to ensure that therapeutic goals are achieved. Dose accumulation also allows us to relate delivered dose to clinical outcomes when evaluating the clinical effectiveness of any ART intervention. In MRgART, plan adaptation on MRI instead of CT adds an additional layer of complexity to the dose accumulation process because the pixel values of MR images do not contain electron density information needed for dose calculation and are subject to signal intensity fluctuations depending on coil setup, magnetic field inhomogeneities, and other factors. Currently, a wide range of research-grade solutions are available for dose accumulation (11–16), but few have been thoroughly validated for clinical use and/or implemented into commercial systems, and none have been specifically optimized for MRgART.

Thus, the development of a robust and accurate dose accumulation solution for MRgART is a subject of active research, particularly within the MR-Linac Consortium (17). In this article, we review and discuss the current status, practical challenges, and potential role of dose accumulation for MRgART and outline a framework for quality assurance of proposed solutions.

## 2 Definition and clinical relevance of dose accumulation

“Dose accumulation” is a term that encompasses a range of techniques for summing multiple RT dose distributions for a single patient (18, 19). The goal is to arrive at a better estimate of the delivered dose compared to standard RT practices, in which a single plan is generated and the dose is calculated only on a static pre-treatment representation of the anatomy. Dose distributions in conventional RT are modeled on the often-flawed assumption that the tumor and surrounding anatomy remain static throughout the course of RT. While it is well understood that the pre-treatment *planned* dose is not equal to the true *delivered* dose due to both RT-induced anatomical changes over time and positional uncertainties during each RT fraction, our current treatment plan acceptability criteria and clinical outcome models are based only on the planned dose (19). Dose accumulation can help us arrive at a more realistic depiction of the delivered dose, but uncertainties in the dose accumulation process make the calculated dose distributions, at best, *estimates* of the delivered dose. Nonetheless, dose accumulation remains a vital mechanism for quantifying delivered dose and evaluating the benefit of various ART strategies used in clinical practice.

Dose accumulation is most often considered in the context of ART, where adaptive plans are generated on either on-board setup images (i.e. on-line ART) or simulation images acquired throughout RT (i.e. off-line ART). In a general dose accumulation pipeline, the dose distribution for each plan is scaled to the number of fractions delivered, the planning images are co-registered, and the dose distributions are then mapped according to the estimated displacements and added voxel-by-voxel (12, 16, 20). Dose accumulation most often utilizes deformable image registration (DIR), which creates a spatial correspondence between two images that accounts for anatomical deformations (21). Doses may be mapped backward onto the pre-treatment anatomy or forward to any time point during treatment, depending on the intended use case for the accumulated dose. Furthermore, dose accumulation techniques may be classified as either inter-fraction or intra-fraction approaches. Inter-fraction dose accumulation uses only the setup image at each fraction (12, 20, 22, 23), while intra-fraction methods account for motion during beam delivery using continuous or periodic motion monitoring imaging such as cine MRI (11, 14). Despite the broad range of methods to perform dose accumulation, we will limit our discussions in this paper to inter-fraction DIR-based methods, as, presumably, any multi-fraction treatment regimen would profit from accurate serial dose estimation. Finally, dose accumulation can be performed either as dose back projection onto the pre-treatment anatomy or forward projection onto the anatomy at any time during or after RT. Both scenarios will be discussed in this article, but the general steps of a dose accumulation workflow remain the same.

Dose accumulation is valuable from a clinical standpoint for a number of reasons, both during and after RT. MRgART allows physicians to set complex goals for treatment personalized for each individual patient, and dose accumulation helps us determine whether the intended goals are being met. These intentions may include sparing dose to specific organs at risk (OARs), escalating dose to target structures, or modifying target volumes as the tumor shrinks to spare tumor-adjacent OARs (24). During a course of treatment, the ability to accumulate dose informs clinicians as to whether or not the daily dose distributions are representative of the cumulative dose. For example, if the dose to an OAR exceeds tolerance on one day, it is clinically relevant to know whether the cumulative dose is in excess, as this may inform the optimization strategy for subsequent fractions (25–27). Alternatively, if OAR doses are sufficiently low after a certain number of fractions, the physician may choose to increase the target dose or add an extra fraction based on individualized treatment response (28–30). When any adaptive modification is made during a course of RT without using a validated dose accumulation method to accurately quantify total delivered dose, potential risks to the patient include overdosing OARs and underdosing target volumes (thus increasing the risk of incomplete treatment response or cancer recurrence). Dose accumulation can enable us to evaluate the safety and efficacy of various ART strategies, which will allow clinicians to personalize RT for each patient while maintaining our field’s commitment to safe, evidence-based treatment approaches.

Dose accumulation may also lead to opportunities to reevaluate normal tissue complication probability (NTCP) and tumor control probability (TCP) models (31–35). The existing dose-response models are largely based on doses calculated on the pre-treatment simulation anatomy, which is often assumed to be static. If we can more accurately quantify the delivered dose after the conclusion of RT in a systematic way, there is an opportunity to refine the current NTCP and TCP models and develop a novel set of planning constraints for the era of ART and personalized medicine (18, 36).

## 3 Considerations for dose accumulation with on-line MR-guided adaptive RT

There are currently two MR-linac systems commercially available: the Elekta Unity (Elekta AB; Stockholm, Sweden) and the ViewRay MRIdian (ViewRay, Inc.; Cleveland, OH, USA). The Unity system combines a modified 1.5 T Philips diagnostic MRI scanner (Philips Healthcare; Best, Netherlands) with a 7 MV flattening filter-free (FFF) linac, while the MRIdian system uses a 0.35 T MRI with a 6 MV FFF linac. Although the specific workflows of the two systems differ, both systems are capable of on-line ART by registering a prior reference (i.e. planning) image to the daily setup image and adapting the reference plan. The MRIdian workflow offers the choice between adapting the reference plan to the current anatomy and delivering the reference plan without modification (8). After the reference and setup images are registered, the user views the predicted dose of the reference plan on the current anatomy and decides whether to treat with the reference plan or adapt. In contrast, the Unity workflow provides two workflow options: Adapt to Position (ATP) and Adapt to Shape (ATS) (9). ATP virtually accounts for the isocenter shift by rigidly registering the reference and setup images but recalculates the dose on the reference image (which can be either a CT or MRI), while ATS involves a full plan adaptation on the setup image (MRI). In either workflow, the original multileaf collimator segments and monitor units can be kept the same to deliver the reference plan without modification.

Although the end-to-end MRgART process differs among MR-linac platforms, these workflows can be summarized as three general classes of solutions: i) treat with the initial plan (e.g. ViewRay MRIdian workflow or Elekta Unity workflows), ii) shift the reference plan to a new position (virtual isocenter shift) and recalculate the dose on the reference plan anatomy (e.g. Elekta Unity ATP workflow), and iii) perform a full re-optimization of the treatment plan on the anatomy of the day (e.g. ViewRay MRIdian workflow or Elekta Unity ATS workflow).

In the absence of commercially available dose accumulation tools that can be run in parallel with the on-line MRgART workflows, there is currently no standardized mechanism for summing and tracking delivered dose over the course of RT. In this section, we will outline a general framework for a potential dose accumulation process for on-line MRgART and discuss considerations and challenges for each step.

A possible workflow for an inter-fraction DIR-based dose accumulation for on-line MRgART would include the following four steps:

1. Autosegmentation of daily setup images for electron density mapping (optional)
2. Recalculation of adaptive plan doses on daily images (optional)
3. DIR between daily images & reference image
4. Deformable dose mapping and dose summation

**Figure 1:**
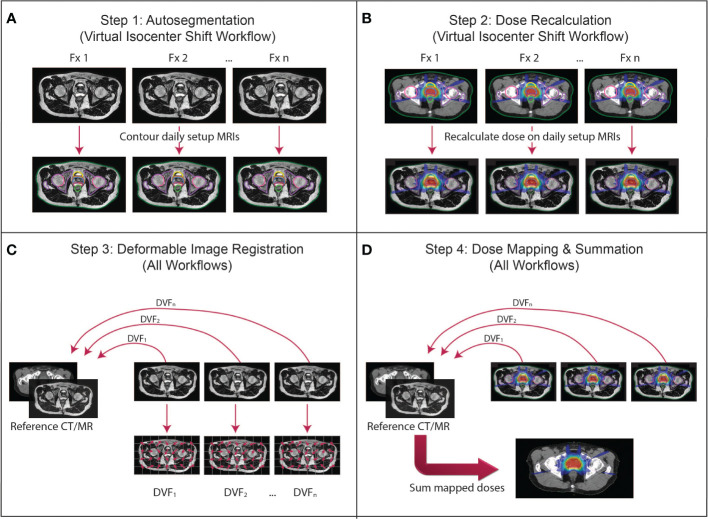
The proposed dose accumulation framework for MRgART. **(A)** Step 1 (virtual isocenter shift workflow only): Because the virtual isocenter shift workflow calculates dose on the reference image rather than the daily setup MRI, the daily images are not contoured during the treatment and must be segmented off-line. **(B)** Step 2 (virtual isocenter shift workflow only): The doses for each fraction must be recalculated on the daily setup images off-line to reconstruct the delivered dose at each fraction. **(C)** Step 3 (all workflows): The geometric correspondence between each daily image and the reference image set (i.e. simulation or any other established time point) is created *via* deformable image registration (DIR) and represented by a deformation vector field (DVF). **(D)** Step 4 (all workflows): The DVFs are applied to the corresponding dose distributions to map the doses onto the reference image set, then the doses are summed to calculate the final accumulated dose. (This figure describes a general workflow where a transformation is established that links the daily image to the reference image through a DVF for the purpose of dose mapping. The details of the implementation depend on the DIR algorithm and mechanism for mapping the dose. The DVFs in this figure are demonstrating the direction of the mapping from the daily images into the reference frame. Whether the dose is “pushed” or “pulled” and whether an inverse DVF is required depend on the implementation of the algorithm created by the user).

For each step of the general framework (**Figure 1**), there may be multiple techniques that may be used to accomplish the same goal, each of which must be thoroughly evaluated for each anatomical site and application. (Depending on the on-line adaptive workflow used, steps 1 and 2 may or may not be necessary; they are most relevant in a virtual isocenter shift workflow where adaptive plan doses are calculated on the reference image rather than daily image. In this case, the delivered dose can be calculated on the daily setup image by segmenting the image to produce an electron density map and recalculating the dose.) We present many of the common approaches that are being investigated for each dose accumulation step as well as considerations for each technique in the realm of MRgART.

### 3.1 Step 1: Autosegmentation of daily setup images for electron density mapping (optional)

To generate an adaptive treatment plan on MRI, the target volume(s) and OARs must be segmented on the image used for dose calculation to create planning constraints and to approximate an electron density map for dose calculation on MRI. Depending on the on-line MRgART workflow used, an autosegmentation step may be necessary for electron density mapping to accurately reconstruct the delivered dose on the daily images prior to implementing a DIR-based dose accumulation approach. This step is particularly relevant in on-line workflows where the daily image is not contoured and the dose is calculated on the reference image rather than the daily image (e.g. virtual isocenter shift workflow) (9). Otherwise, if segmentation is done during the on-line workflow, this step would not be necessary.

Several autosegmentation methods are appropriate for MRgART, including DIR-based contour propagation from the reference image as well as atlas-based and deep learning approaches. In the first method, DIR is used to generate a spatial correspondence between the daily image and the reference image or some other prior image, and the contours are mapped according to the established transformation (37). The quality of the segmentations depends on the DIR algorithm performance; specific considerations for DIR with MRgART are discussed in Step 3 below.

Next, atlas-based autosegmentation uses a small collection of contoured image sets (i.e. “atlases”) which serve as templates for contouring the image set of interest (i.e. “patient”) (38). Each atlas is aligned with the patient, and contours are propagated *via* DIR to produce one structure set per atlas on the patient. These intermediate results are combined into a final structure set using either a method to combine all structure sets such as a STAPLE algorithm (38–40) or a voting mechanism which selects the best contour for each structure such as Majority Vote (38, 41) or Random Forest (42, 43). Atlas-based approaches have historically used atlases from other patients (i.e. “inter-patient”), but serial imaging for on-line ART introduces the possibility of using a patient’s own images from prior fractions as atlases (i.e. “intra-patient”) (44). Using multiple intra-patient atlases has the potential to improve performance over both inter-patient atlas-based approaches and intra-patient DIR from the reference plan image—especially for later fractions when anatomical changes from the simulation image may be quite large—but requires further validation. Intra-patient methods can also produce comparable performance to inter-patient methods with fewer atlases, which speeds up execution time considerably.

Another promising autosegmentation approach, deep learning, uses a large number of contoured image sets to train a computer algorithm called a deep neural network to contour unlabeled input images (45–47). Most deep learning autosegmentation methods use a convolutional neural network architecture such as U-Net (48), which is formed by stacking multiple hidden layers, including convolutional layers, that each learn a feature of the training data. After a model is trained and validated, it can be used to contour unseen input images at rapid speeds. While atlas-based approaches typically reach peak performance using 5-15 atlases (49–52), deep learning generally requires dozens to hundreds of images as training data for optimal performance (53, 54). As more patients are treated on MR-linac devices with standardized MR sequences, we can leverage vendor-supported data repositories such as the MOMENTUM study (55) to aggregate curated, high-quality imaging data in a coordinated, efficient manner to train these models (56).

There are a few nuances to autosegmentation on MR rather than CT. MR demonstrates enhanced soft tissue contrast, rendering many OAR boundaries more clearly visible on MR than on CT and thus potentially improving the quality of autosegmentation (57). However, unlike CT, pixel values in T1- and T2-weighted MRIs are not inherently quantitative and are subject to variations due to coil positioning, radiofrequency and electronic noise, and magnetic field inhomogeneities (58–60). The arbitrary pixel value scaling and image intensity variations may affect the performance of autosegmentation algorithms, which rely on intensity and/or contrast similarities between training data/atlases and the image sets to be segmented. These effects can be minimized on the acquisition side by using consistent pulse sequence parameters, immobilization, and coil setup during MRgART and on the post-processing side by performing image intensity standardization on the images (61, 62). Furthermore, while one advantage of MR is the wide array of image contrast mechanisms obtained by using different pulse sequences, each of the aforementioned autosegmentation techniques is pulse sequence-specific, meaning that they should be trained and executed on images with identical pulse sequence parameters for optimal performance (63, 64). However, aggregating data across different patients and/or institutions for deep learning models will require adoption of consistent protocols across sites or the implementation of data augmentation techniques to generalize trained models to multiple sequences (65, 66).

### 3.2 Step 2: Recalculation of adaptive plan doses on daily images (optional)

Depending on the workflow used, the dose distributions for adaptive plans may need to be recalculated on the daily setup image off-line prior to accumulating the doses. Like Step 1, this step would be required if the dose is calculated on the reference image rather than the daily setup image during on-line plan adaptation (e.g. virtual isocenter shift workflow).

Unlike CT, where the pixel values represent physical measurements of photon attenuation in Hounsfield units (HU) and are easily converted to relative electron density (ED) maps to calculate dose, dose calculation on MRI requires approximation of the ED values for each voxel. Currently, the most common ED approximation method is called bulk density assignment: for each structure contoured on the planning CT, the mean ED value of the structure on CT is assigned to all voxels in the structure on MRI (8, 9). These values may be overridden with user-defined values when corrections are needed or if no CT is available in an MR-only RT workflow. Bulk density assignment has been shown to be accurate across disease sites, resulting in minimal deviations in dose volume histogram parameters compared to doses calculated on CT (37–39), but may not adequately handle largely heterogeneous volumes such as spinal vertebrae (40), femoral heads (39), or lung (41). An alternative approach is to deformably register the planning CT into the MR frame of reference to create an ED map (42, 43). While this method preserves the heterogeneity of ED values throughout each structure, CT-to-MR DIR may have limited accuracy due to the different signal and contrast characteristics of the two modalities (44, 45), and it will likely fail in situations with large deformations such as lung collapse.

As researchers move toward MR-only treatment planning, alternative ED approximation methods are also being explored that do not require any CT input and would eliminate the need for segmentation of daily MRIs for ED mapping (46). Synthetic CTs can be generated directly from MRIs using specialized MR sequences such as Dixon MRI, which separates signal from fat and water and enables clusters of voxels to be assigned discrete tissue classes with associated ED values, similar to the bulk density assignment (47, 48). Another option is to apply a calibration curve relating MRI signal intensities to HU values on a voxel-wise basis to generate a synthetic CT that preserves tissue inhomogeneities (49, 50). This method can be used directly on the T1- or T2-weighted images used for setup and plan reoptimization rather than requiring an additional MR sequence. However, MR pixel values are subject to intensity variations, and depending on the contrast mechanism of the pulse sequence, the calibration curve is unlikely to follow a simple linear or logarithmic fit. Deep learning models may also be trained to generate heterogeneous synthetic CTs from Dixon or T1- or T2-weighted MRIs. Several studies have demonstrated excellent performance of such models (51–54).

It should also be noted that a shift invariance approach, which involves a simple shift of the dose distribution to account for the isocenter shift, may be used instead of a full dose recalculation. This approach has been shown to be a good approximation of the delivered dose for many deep lying tumors but fails for shallow tumors in the build-up region and when anatomical changes are substantial (55, 56).

### 3.3 Step 3: DIR between daily images & reference image

Once the dose distributions for each adaptive plan have been accurately calculated on each daily setup image, the next step is to establish a geometric transformation between each daily image and the reference image *via* DIR. This step of dose accumulation is required regardless of the on-line MRgART workflow used.

There are several DIR approaches with varying degrees of complexity, which are summarized in the literature (57–59). Most implementations of DIR share three main components: 1) a transformation, or a mathematical model, that establishes the geometric correspondence between the source and target images; 2) an objective function, which typically includes a similarity metric used for evaluating the alignment between the images and a regularization term to impose constraints on the deformation field; and 3) an optimization method that optimizes the parameters of the transformation model to maximize the similarity between the source and target images under the imposed constraints (57–59). In this section, we will focus on the first two elements and their implications for dose accumulation in MR-guided adaptive RT.

A number of transformation models are available for DIR, including B-spline and several non-parametric models. The B-spline transformation is a commonly used non-linear parametric model generated using a weighted sum of a set of spline functions defined at a set of control points spaced evenly throughout the source and target images (21, 57, 60). In contrast, non-parametric models such as elastic, fluid and optical flow and finite element methods can generate much more complex transformations to model anatomical changes (58, 59, 61–64). Unlike B-spline models, which represent the image deformation using parameters defined at each control point, non-parametric transformation models are usually represented by more complex deformation vector fields (DVFs) where the displacement in all three directions is defined for each individual image voxel. When selecting a transformation model to use for dose accumulation, one must consider the expected degree of deformation in the anatomical site of interest as well as the complexity and underlying assumptions of the model. This choice is particularly important for dose accumulation, as the transformation model impacts the registration accuracy within contrast-devoid regions and, implicitly, the accuracy of the accumulated dose.

The second component of any registration algorithm is the similarity metric, which is used to evaluate the alignment between the registered images throughout successive iterations in the optimization process. Similarity metrics are classified as either intensity-based or feature-based (58, 59). Intensity-based metrics evaluate the alignment of intensity patterns (i.e. gray-scale information) between the source and target images. Feature-based metrics use anatomical landmarks such as points, lines, and surfaces to obtain the correspondence between the source and target images.

The choice of similarity metric depends on the intensity ranges and modalities of the source and target images. In the context of MR-guided adaptive RT, intensity-based metrics such as sum of squared intensity differences (65) and/or cross correlation (66) typically work well for images of the same modality and intensity range, such as the daily MRIs, as long as the same MRI pulse sequence is used for setup at each fraction of a patient’s treatment. Registration across modalities such as CT-to-MR and registration across different MR sequences such as T1-to-T2 present a more complex problem due to intensity inconsistencies between the images. Despite the different contrast mechanisms and intensity ranges, most of the recently developed multi-modality registration approaches still use certain intensity-based metrics such as normalized mutual information (67) over feature-based metrics. Normalized mutual information is based on global histogram matching (i.e. the distribution of intensity values across the entire image). Several alternative approaches have been proposed for multi-modality registration, including normalized gradient fields and modality independent descriptors. The normalized gradient fields metric uses the gradient (i.e. derivative) of the intensity in each image rather than the image intensities themselves (68, 69). In the modality independent descriptors approach, the images are pre-processed into a modality-independent format that preserves local image feature information and can be directly compared using established similarity metrics (70, 71).

While the field of multi-modality DIR has made great progress in recent years, many of these newer techniques have yet to be implemented into commercial treatment planning systems for MRgART. Recent studies have shown that the CT-to-MR registration currently used for the 1.5T MR-linac workflow underperforms compared to same-sequence MR-to-MR registration in both prostate (45) and head and neck cancers (72). This discrepancy has implications for daily plan adaptation and dose accumulation. Many clinics create the reference plan on the CT simulation, while others acquire a CT for electron density information but create the reference plan on the MR simulation. The latter method may improve the quality of the DIR in the on-line workflow, which will likely reduce the time spent manually editing contours. For dose accumulation, if doses are mapped back to the pre-treatment time point for comparison to the reference plan, the DIR quality may improve if the daily MRIs and daily fraction doses are registered to the MR simulation image or first fraction MRI rather than the CT. However, the implementation of state-of-the-art algorithms may improve the performance of multi-modality image registration, and rigorous evaluation of both CT-to-MR and MR-to-MR DIR quality is needed for all organ sites.

In addition to the similarity metric, DIR algorithms must impose constraints on the deformation field by adding a regularization term to the objective function. When a regularization term is used, the final solution (i.e. the estimated deformation field) will be a tradeoff between maximizing the similarity between the source and target images and satisfying the constraints. Some examples of constraints include preserving topology, ensuring a smooth deformation field, and penalizing non-physical deformations given prior knowledge of the underlying anatomy (e.g. preventing the warping of rigid structures such as bones) (59, 73). In anatomical regions with sliding tissues such as the lung and chest wall, regularization terms that allow a discontinuity in the DVF can be used (74, 75). Another constraint with particular relevance to dose accumulation is inverse consistency, which ensures that the forward and backward transformations, computed simultaneously, are direct inverse mappings of one another. Inverse consistency would be an important consideration if clinicians are interested in evaluating accumulated dose in both the forward and backward directions. (See “Interpretation of Accumulated Dose” section for a more detailed discussion on forward and backward mapping.)

While the myriad of deformable image registration algorithms enable us to model anatomical changes in a wide range of clinical scenarios, these algorithms are all based on fundamental assumptions about the anatomy that do not always hold true throughout a course of RT. For example, assumptions that the deformations are smooth/continuous and invertible are violated in scenarios such as organ sliding and tissue gain/loss. While these assumptions are necessary for estimating deformations in an anatomically plausible matter, they also fundamentally limit the ability of DIR to accurately characterize the true anatomical changes.

An additional consideration for DIR in the context of dose accumulation for daily MRgART is that image sets from up to dozens of fractions will need to be registered, but image registration occurs as a separate operation between only two sets of images. If all doses are being mapped back to the simulation image, the simplest options for composing registrations are 1) registering each daily image to the reference image, or 2) registering each image onto the previous fraction’s image in a sequential fashion to create a “DVF chain” (76). The second option is particularly advantageous when anatomical changes between the beginning and end of the course of RT are substantial because it minimizes the change between each image set being registered. However, if the performance of the DIR algorithm is poor despite the minimal anatomical change from day to day, this approach compounds the error throughout the chain of registrations and dose deformations (76).

### 3.4 Step 4: Deformable dose mapping and dose summation

Once the daily image sets are registered, the doses are mapped by applying the transformations defined by the image registrations to the dose grids. If the doses of each adaptive plan are scaled to the prescription dose throughout the entire course of RT, then they must be scaled down to the dose delivered at each fraction. Finally, the mapped doses are summed voxel-by-voxel to calculate the delivered dose distribution. (If large variations exist in the dose delivered to each structure from day to day, fractionation effects should also be taken into account using linear quadradic models.)

While this process is, in effect, a simple computational task after the DVFs between each corresponding image set have been calculated, the discrete nature of voxels/dose grids and the deformations occurring between each time point make it infeasible to assume that the same individual cells are contained within the same matched voxels from day to day. For this reason, tri-linear interpolation is often employed: each voxel is divided into sub-volumes before dose mapping, and the values of the mapped sub-volumes corresponding to the same voxel on the reference dose grid are averaged and assigned to that voxel (21, 77, 78). The interpolation method is fast, which may be highly advantageous in the context of MRgART when there is a different treatment plan for each fraction. However, this method is less accurate in steep dose gradients and treats dose as an imaging voxel intensity rather than a physical quantity (i.e. energy per unit mass) (79, 80).

It’s important to note that the details of the algorithm that performs the DIR and dose mapping will specify the direction of the DVF and whether the dose mapping is done by “pushing” the dose from the image it’s calculated on to the summed image or “pulling” it. In many scenarios, one direction of the DVF is sufficient for DIR and dose mapping, but how that is implemented depends on the specifics of the algorithm. The combination of the DIR algorithm and dose mapping mechanism will dictate whether an inverse DVF is required.

While this issue is not unique to MR-guided therapy, no discussion on dose accumulation would be complete without mentioning the difficulties in accurately accumulating dose when tumors or OARs exhibit volumetric changes throughout RT. Conceptually, one must consider what happens to the dose delivered to a small volume of tissue if that tissue disappears before the end of treatment, which routinely occurs for certain tumor types such as human papillomavirus-positive oropharyngeal cancers (16). This dilemma is illustrated mathematically by Zhong & Chetty (79), who demonstrate that simply deformably mapping dose violates the fundamental physics principle of conservation of energy. Energy/mass transfer methods have been proposed to account for conservation of energy, whereby the energy deposited in each voxel and the mass of each voxel are mapped separately onto the reference dose grid then divided to calculate the dose (21). The initial implementations of energy/mass transfer-based dose accumulation used Monte Carlo methods to simulate the energy deposition in each voxel (81, 82), which required significant computational power. In more recent years, these approaches have been extended to non-Monte Carlo techniques that can be interfaced with commercial treatment planning systems (83) and run in real-time (84).

Finally, it is important to remember when designing a dose accumulation workflow that translation of the couch after the image acquisition must be incorporated into the dose accumulation process. The MR-linac on-line treatment adaptation workflows already account for this through either a physical couch shift (ViewRay MRIdian) or a virtual isocenter shift (Elekta Unity). However, if images and doses are exported outside of the closed system of the MR-linac device and its associated treatment planning system, one must ensure that the dose accumulation algorithm accounts for the isocenter shift prior to dose mapping.

## 4 Validation and quality assurance of DIR for dose accumulation

### 4.1 General considerations

To use DIR within the dose accumulation workflow, the employed DIR algorithms must fulfill particular validation benchmarks and must be subjected to stringent quality assurance (QA) criteria to ensure patient safety and the attainment of the therapeutic endpoint. A distinction has to be made, however, whether the aim is the commissioning of a DIR solution prior to clinical use or whether the QA of the estimated deformations at the time of treatment needs to be ensured. Depending on the situation, different criteria may be considered in favor of others. For example, criteria which require known inputs such as expert contours, landmarks and/or deformations are more suitable for commissioning, whereas QA at the time of treatment preferably relies on criteria with a higher degree of automation. In this section, we will briefly describe several classes of criteria which can be used for this purpose, while, where applicable, also indicating value ranges for these criteria where DIR algorithms may be considered reliable. It is important to note, however, that while the discussed criteria provide a practical starting point for evaluation of DIR, acceptability criteria for any metric are necessarily driven by specific application considerations. A summary of the criteria discussed below and recommended tolerances are provided in **Table 1**.

**Table 1:** Summary of quality assurance metrics for deformable image registration (DIR) and recommended tolerances.

Metric	Tolerance/Ranges	Reference
**Dice Similarity Coefficient**	Structure size-dependent (~ 0.8 – 0.9)	(73, 85)
**Jaccard Index**	Structure size-dependent (~ 0.8 – 0.9)	(86–88)
**Hausdorff Distance,** **Mean Distance to Agreement**	Maximum voxel size (~2 – 3 mm)	(73, 85)
**Target Registration Error**	Maximum voxel size (~2 – 3 mm)	(73, 85)
**Distance to Discordance Metric**	Maximum voxel size (~2 – 3 mm)	(89, 90)
**Jacobian Determinant**	Tissue-dependent (~0.8 – 1.2 for biological soft tissues)	(73, 91, 92)
**Curl Magnitude**	Tissue-dependent (~0 – 0.2 for biological soft tissues)	(91, 93)
**Biomechanical Criteria with Thresholds on Mechanical Stresses**	Tissue-dependent	(91)
**Dosimetric criteria**	Application-dependent	(94–97)

### 4.2 Qualitative criteria

A simple approach for both validation and QA of DIR is visual inspection of the post-registration alignment of organ boundaries and/or high-contrast anatomical landmarks (73, 85, 98). This can typically be performed by the radiation oncologist, physicist, and/or radiation therapy technologist as soon as the registration step is completed and can help identify gross potential mis-registrations. For same-contrast images, this may be complemented by a visualization of intensity-based maps such as the absolute image difference or structural similarity (99–101), which contain brighter or darker voxels, depending on the degree of alignment between the registered images. However, visual inspection is typically subject to interpretation, and two registrations can be visually identical while having completely different anatomical mappings. While visual assessment of the DIR result is necessary, it alone is not sufficient, and therefore additional objective complementary criteria are required.

### 4.3 Contour-based criteria

A feasible solution towards the commissioning of DIR is the evaluation of the algorithm’s capability for aligning organ boundaries. If a DIR algorithm consistently fails to provide a satisfactory boundary alignment, then that is a good indicator that its performance may be insufficient for an accurate dose accumulation. In this sense, the Dice similarity coefficient (DSC) (73, 102, 103) and the Jaccard index (103, 104) provide an objective manner of evaluating an algorithm’s capability for organ boundary alignment. Using the DSC and the Jaccard index as a DIR validation criterion requires expert-drawn contours of the same anatomical structure(s) on the images to be registered for ground truth comparison. The DIR-estimated deformations are used to map the contour(s), and the two criteria can be used to evaluate the overlap between the expert-drawn and the DIR-mapped contour(s). The values of both the DSC and the Jaccard index range from zero to one, with zero indicating no overlap and one corresponding with perfect overlap. In the scope of image-guided radiotherapy, a DIR algorithm that provides consistent DSC and Jaccard index values of 0.8 – 0.9 is generally considered to be reliable (73, 85–88). However, the values of the DSC and Jaccard index depend heavily on the volume of the structure; a large structure such as brain could have a DSC or Jaccard index close to 1 and a small structure such as the optic chiasm close to 0 for the same geometrical distance. Therefore, tolerance values for DSC and Jaccard index should be based on structure size and cannot be generalized.

In addition to the DSC and the Jaccard index, complementary criteria such as the Hausdorff distance (HD) (103, 105) and the mean distance to agreement (MDA) (73, 86, 103) are also recommended for inclusion. Instead of evaluating the post-registration contour overlap, the HD and the MDA are used to compute an actual distance between the mapped and the expert-drawn contours, providing further information on the validity of the DIR-estimated deformations in the vicinity of organ boundaries. Acceptable values for both the HD and MDA should be within the uncertainty of the contouring process, typically in the 2 – 3 mm range (73, 85). However, it should be noted that the HD represents the maximum distance between associated boundary points in the contours and is therefore more prone to outliers than the MDA; alternatively, the 95% HD, which reports the 95^th^ percentile of distances between boundary points, can be used instead to limit the effects of outliers.

As previously stated, criteria such as the DSC, Jaccard index, HD and/or MDA provide an evaluation of the organ boundary alignment and/or volume overlap capabilities of DIR algorithms. However, they are limited in their ability to provide a comprehensive evaluation of the estimated deformations because they provide no information about the accuracy within organ boundaries. Moreover, criteria such as the DSC and the Jaccard index are strongly dependent on the size of the evaluated contours and may thus lead to an interpretation bias. On the other hand, it has to be taken into consideration that the MR-Linac allows for a more accurate definition and delineation of anatomical structures due to the high soft tissue contrast present in the MR images. This is a considerable advantage in areas containing a large number of small anatomical structures (e.g. head and neck), thus allowing a more consistent evaluation of the DIR algorithm performance *via* contour-based criteria.

### 4.4 Criteria employing known displacements/deformations

The target registration error (TRE) (73, 106) allows a quantitative evaluation of a DIR method’s accuracy and precision in any anatomical region showcasing identifiable anatomical landmarks and is thus not limited to organ boundaries. The calculation of the TRE requires an expert to manually indicate the location of the same anatomical landmarks on the images to be registered. The DIR-estimated deformations are then used to map the landmarks annotated on one of the images onto the second image. The distance between the mapped and the manually annotated landmarks on the second image is then calculated to evaluate the registration errors. For a DIR algorithm to be considered reliable for clinical use, the average TRE calculated for the annotated landmarks should consistently reside under the maximum image voxel size, typically in the 2-3 mm range (73, 85). Similar to defining anatomical boundaries, the manual annotation of anatomical landmarks is also facilitated by the high soft tissue contrast provided by MR images. In turn, this may lead to an improved landmark-based evaluation of a DIR solution compared to imaging modalities such as CT/CBCT, in the absence of contrast administration. Still, while such an approach can aid in evaluating the typical accuracy and precision of DIR methods in the vicinity of high-contrast anatomical landmarks, it has limited validation capabilities for homogeneous image areas due to the intrinsic difficulty of defining and annotating landmarks in such regions. Also, many landmarks are defined at extreme positions of an organ and can therefore represent different anatomy if the organ slides or rotates (107, 108). Moreover, the landmarks themselves are often used by the data similarity term of the DIR algorithm and therefore may not be a completely independent measure.

These limitations can be addressed, for example, by the use of physical (96, 109–111) and/or digital phantoms (92, 111, 112). Physical phantoms typically consist of tissue mimicking materials which are displaced/deformed in a controlled manner under the effect of a mechanical actuator (e.g. a piston) such that the displacements/deformations of the phantom are (partially) known (potentially by the use of implanted fiducials). While physical phantoms which can be effectively and safely operated within an MR-Linac are commercially available, the design, development and optimization of MR-visible phantoms is an ongoing area of investigation.

### 4.5 Criteria based on tissue biomechanics

The QA of DIR algorithms can also be performed by employing criteria based on the mechanical properties of the observed anatomy (87, 91). Depending on individual physical properties, the deformations of the various anatomical tissues have a limited number of degrees of freedom. For example, elastic biological soft tissues are near-incompressible due to their high water content, and therefore, strong compressions and expansions within such regions are anatomically implausible. In effect, if such implausible deformation patterns are estimated by the employed DIR algorithm, a misregistration has most likely occurred.

To determine the amount of compression or expansion present in the estimated deformations, a voxel-wise evaluation of the Jacobian determinant of the deformations can be performed (73, 92). It is known from continuum mechanics that the Jacobian determinant of incompressible materials is equal to 1. Thus, large deviations from 1 within the deformations estimated for elastic biological soft tissues are indicative of misregistration. Similarly, during typical anatomical motion, strong local torsions are not expected to occur deep within the boundaries of elastic soft tissues and can also indicate the occurrence of misregistration. Such torsions can be quantified, for example, by evaluating the curl magnitude of the deformations, with large local deviations from zero being anatomically implausible (93). Typical values of these metrics for the liver and kidneys have been determined to be between 0.8-1.2 for the Jacobian and 0-0.2 for the curl magnitude (91). We do not expect the deformations within other elastic soft tissues to deviate significantly from these values.

Alternatively, QA criteria relying on the biomechanical properties of the observed anatomy can be even further individualized for specific tissues. By providing the elastic modulus and Poisson ratio of the structures of interest as input during the planning/re-planning phase of treatment, the mechanical stress occurring as a result of the estimated deformations can be evaluated within these regions (91). The two parameters can be extracted either from look-up tables, following mechanical tests performed in previous studies, or from quantitative functional imaging such as MR elastography (113). During typical anatomical motion, the mechanical stress within the observed tissues is not expected to lead to tissue rupture or occlusion of blood circulation. Therefore, if such occurrences are detected within the DIR-estimated deformations, they are most likely indicative of misregistration and have been shown to be correlated with errors within the accumulated dose map (91). Such tissue-damaging mechanical stress limits are tissue-specific and can again be extracted from look-up tables generated on the basis of previous studies which have performed the required mechanical tests.

### 4.6 Dosimetric criteria

While it is generally agreed that geometric DIR uncertainties play a determining role in the accuracy of deformable dose accumulation, the precise manner in which such uncertainties relate to dose accumulation errors is the topic of ongoing research. For example, DIR errors within isodose areas will most likely have less of an impact on the overall accumulated dose compared to registration errors occurring within regions containing steep dose gradients. In this sense, previous studies propose establishing a non-linear relationship between the DIR and the warped/accumulated dose uncertainties (94, 95). The challenge is hereby the selection of the criteria used to evaluate the DIR accuracy as well as the dosimetric parameters to be used as an input for the non-linear model. An additional challenge is determining the model itself, which could, for example, imply an empirical selection of a composition of a set of mathematical functions or an estimation of the model by means of machine learning. An alternative approach, which bypasses the construction of such a model, consists of the use of deformable phantoms made of radiosensitive gels (96, 97). The design paradigm for such phantoms is similar to the one used for phantoms evaluating DIR uncertainties (as described above) with, for example, the additional inclusion of radiosensitive materials whose MR signal is dependent on the absorbed radiation dose. Irradiating such phantoms while undergoing deformations, followed by an MR-based readout of the delivered dose, provides a theoretical gold standard for the warped dose, which can be subsequently compared to the one estimated by the DIR algorithm. For the purpose of commissioning DIR algorithms for dose accumulation, this provides a direct estimation of the expected dose accumulation errors of the algorithm under evaluation. However, it is important to take into consideration the high sensitivity of existing gels to environmental factors such as temperature, as well as phantom deformations in the absence of irradiation. This can in turn introduce uncertainties within the readout process and therefore bias the evaluation of the DIR algorithm. Consequently, the construction of robust radiosensitive phantoms is the subject of ongoing research.

## 5 Interpretation of accumulated dose

**Figure 2:**
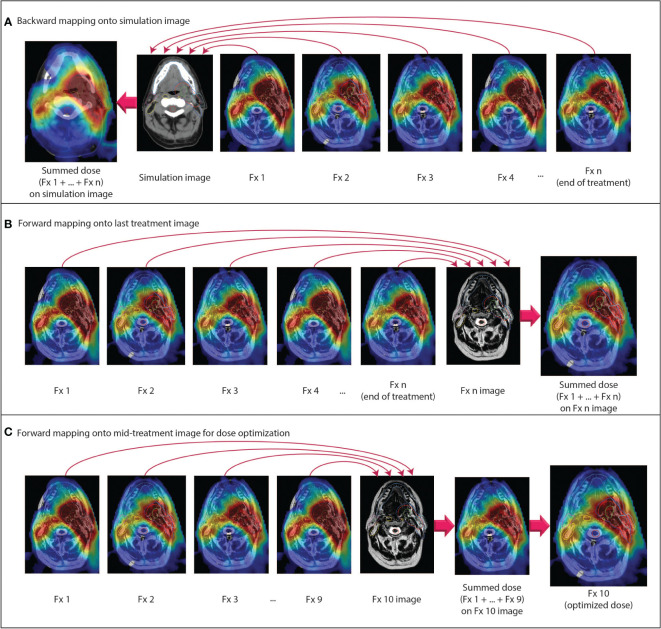
Examples of different dose accumulation perspectives. **(A)** Doses from all fractions are mapped backward onto the pre-treatment simulation anatomy. **(B)** Doses from all fractions are mapped forward onto the anatomy from the last fraction. **(C)** At a given time point during RT, all previous doses are mapped forward onto the anatomy for the current fraction. The summed dose is used as an input for dose-optimized adaptive replanning. All doses are scaled to the total prescription dose.

For simplicity, we have assumed until this point that the dose distributions from each adaptive plan are always mapped back onto the reference (i.e. simulation) image for comparison between the planned and delivered doses. In clinical practice, doses may be mapped either forward or backward to any time point (i.e. simulation, any fraction during RT, or post-RT), as illustrated in **Figure 2**. However, the aforementioned issues in dealing with volumetric changes and tissue gain/loss will inevitably cause differences in the accumulated dose depending on the direction of dose mapping (79). Rather than considering which direction is “more accurate,” we can regard forward- and backward-mapping as two distinct perspectives for understanding accumulated dose, each designed to answer different clinical questions.

When doses are mapped backward and accumulated in the pre-treatment simulation image frame of reference, it is straightforward to compare the planned dose to the accumulated dose over the entire course of RT. For an individual patient, this type of analysis primarily serves to determine whether the intended goals of therapy were met in the aggregate of all adaptive plans. In cases where the intent of adaptive RT was to reduce dose to OARs or escalate dose to the tumor, comparison to the planned dose on the pre-treatment anatomy can also determine whether OAR doses were indeed lowered or whether tumor doses were indeed increased, respectively. One drawback of such a comparison, performed for an individual patient post-RT, is that it is too late to modify the treatment if the intended goals were not met. Still, such comparisons may inform clinicians as to how to approach future patients by establishing a relationship between dose of the day and accumulated dose.

Instead, the post-RT backward-mapped accumulated dose is perhaps a more useful quantity when analyzing side effects and treatment response on large cohorts of patients. Although we have already entered the era of daily MR-guided adaptive replanning, many questions remain regarding the degree of OAR dose sparing and reduction in clinical side effects that can be achieved with this technique, as well as how we can identify the individual patients who stand to benefit the most from treatment on an MR-linac. To answer these questions, we may analyze cumulative delivered dose in the same perspective in which we have historically conceptualized RT: in the frame of reference of the pre-treatment simulation scan. Furthermore, as previously mentioned, the OAR dose constraints and prescription doses that we use for planning are derived from NTCP and TCP models, respectively, which are based on planned doses on the pre-treatment simulation anatomy. In the era of adaptive RT and dose accumulation, more research is needed to determine whether these models remain accurate for delivered dose and whether dose constraints should be redefined in the context of adaptive RT (18, 36).

While this type of post-treatment analysis may be advantageous for answering many research questions, there may still be instances when we want to use a patient’s cumulative delivered dose at some point during RT to adapt the remaining fractions. Backward mapping may be used in this scenario, whereby the accumulated dose over a certain number of delivered fractions can be compared to the proportion of the total planned dose for the same number of fractions. However, if the ultimate intention is to use the accumulated dose to modify the treatment plans for subsequent fractions, it may be more appropriate to map the dose forward into the frame of reference in which the next fraction will be planned.

If forward dose mapping can be automated and integrated into the MR-linac on-line clinical workflow, it may radically change how we approach daily MRgART. In current commercial MR-linac systems, adaptive plans are currently generated *via* warm start optimization using the reference plan as a starting point (9, 13, 114). A previous adaptive plan may be used as a new reference plan rather than the pre-treatment reference plan, but the IMRT objectives and dose constraints remain the same for each adaptive plan unless they are manually modified. We have seen with this process that there is a reasonable degree of dosimetric variability between plans from day to day despite the same objectives being used for planning. For example, even if an OAR dose constraint is violated in one or more fractions, that constraint may still be met in the cumulative delivered dose if doses to that OAR fall far enough below the constraint threshold for all other fractions (72). If we could accumulate the dose at each fraction in the frame of reference of the daily setup image, then we could adapt the plan using modified and/or re-prioritized planning objectives based on knowledge of the cumulative delivered dose. In other words, a dose constraint that is routinely met may be de-prioritized in the set of IMRT planning objectives in favor of a dose constraint that is routinely violated. This approach may also be useful if the clinical intent is to escalate dose to the tumor because the physician can make an informed decision about how much the tumor dose can be increased without exceeding the OAR constraints.

In summary, we propose that dose accumulation for purposes of toxicity assessment should be reported on the planning image, as that represents the anatomy at the time of planning and is necessary for useful implementation of toxicity models. However, for ART purposes, we propose that the accumulated dose should be represented on the most recent anatomy so that replanning can be assessed and applied.

As a final note, as with every step of the radiation therapy process, cumulative dose has an associated uncertainty that is a combination of the uncertainties in each step of the dose accumulation process. Understanding these uncertainties and how they impact the use of the cumulative dose is critical to clinical decision making. Thus, prior to clinical implementation of any dose accumulation workflow, it is essential for clinicians to clinicians to fully understand the process and the inherent uncertainties so that they can make the best decisions for their patients.

## 6 Discussion

High-frequency on-treatment imaging and target volume serial assessment with MR-linac devices now affords clinicians the opportunity to move past the historical concept of *planned dose* into an era where *delivered dose* can be used for individual response assessment and dose-optimized adaptive RT. However, the implementation of dose accumulation requires a basic understanding of key considerations and careful validation of dose accumulation solutions tailored to distinct clinical scenarios. In this review article, we have outlined four general steps for dose accumulation—autosegmentation, dose calculation, deformable image registration, and dose mapping/summation—and discussed considerations specific to MR-linac/MRgART workflows. Additional efforts to standardize best practices are imperative to ensure that we move towards a future of adaptively optimized dose as patient-specific precision radiotherapy evolves.

## Author contributions

All authors participated in discussions regarding the concept/design and topics reviewed in this manuscript. The manuscript was written by BM, CZ, and MN. All authors contributed to the article and approved the submitted version.
